# High expression levels of centromere protein O participates in cell proliferation of human ovarian cancer

**DOI:** 10.1186/s13048-024-01452-x

**Published:** 2024-06-18

**Authors:** Li-hui Si, Guang-chao Sun, Zi-wei Liu, Shi-yu Gu, Chu-han Yan, Jin-yuan Xu, Yan Jia

**Affiliations:** 1 Department of Obstetrics and Gynecology, The second Hospital of Jilin University, Changchun, Jilin China; 2 Department of Neurosurgery, The Second Hospital of Jilin University, Changchun, Jilin China

**Keywords:** Ovarian cancer, Centromere protein O, Cell proliferation, Cell apoptosis, Cell cycle

## Abstract

Ovarian cancer is a common malignant tumor in women, with a high mortality rate ranking first among gynecological tumors. Currently, there is insufficient understanding of the causes, pathogenesis, recurrence and metastasis of ovarian cancer, and early diagnosis and treatment still face great challenges. The sensitivity and specificity of existing ovarian cancer screening methods are still unsatisfactory. Centromere protein O (CENP-O) is a recently discovered structural centromere protein that is involved in cell death and is essential for spindle assembly, chromosome separation, and checkpoint signaling during mitosis. The abnormal high expression of CENP-O was detected in various tumors such as bladder cancer and gastric cancer, and it participates in the regulation of tumor cell proliferation. In this study, we detect the expression abundance of CENP-O mRNA in different ovarian cancer cells ( ES-2, A2780, Caov-3, OVCAR-3 and SK-OV-3). The biological function changes of cell proliferation and apoptosis were detected and the role of CENP-O in ovarian cancer cell proliferation and apoptosis was explored by knocking down the expression of CENP-O gene. The results showed that CENP-O gene was significantly expressed in 5 types of ovarian cancer cell lines. After knocking down the CENP-O gene, the proliferation and cloning ability of ovarian cancer cells decreased, and the apoptosis increased. This study indicates that CENP-O has the potential to be a molecular therapeutic target, and downregulating the expression of CENP-O gene can break the unlimited proliferation ability of cancer cells and promote their apoptosis, providing a foundation and new ideas for subsequent molecular mechanism research and targeted therapy.

**Supplementary Information:**

The online version contains supplementary material available at 10.1186/s13048-024-01452-x.

## Introduction

Ovarian cancer (OV) is one of the most malignant tumors in the female reproductive system with a fatality rate of approximately 47.4% [28]. The main reason for this high mortality rate is the low early diagnosis rate, with about 70% of OV cases being diagnosed at an advanced stage [7]. In 2018, there were 295,414 new cases and 184,799 deaths from ovarian cancer worldwide [34], with the number of affected women increasing in many low- and middle-income countries. Additionally, as life expectancy increases, the number of diagnosed cases is also growing each year. Surgical resection, cell ablation, and platinum-paclitaxel combination chemotherapy are the most commonly used methods for OV treatment [25]. Over the past 30 years, the survival rate of OV patients has increased by about 20%, thanks to continued improvements in OV treatment methods [15]. While surgical intervention and frontline chemotherapy can provide clinical relief for some ovarian cancer patients, the high recurrence rate and low survival rate remain unchanged [31, 34], underscoring the need to further understand the pathophysiological characteristics of ovarian cancer cells.

Studies have shown that vascular endothelial growth factor (VEGF), poly (ADP-Ribose) polymerase (PARP), and the PI3K/Akt/mTOR signaling pathway can promote OV cell proliferation and metastasis [6, 18, 20]. Targeted therapy drugs against these key molecules and pathways have been applied clinically. However, the occurrence and development of OV are regulated by multiple molecular interactions that have not yet been fully elucidated. Therefore, discovering new biomarkers is still crucial for understanding the mechanisms and treatments of OV. In addition to identifying novel biomarkers for early diagnosis, better understanding of the molecular mechanisms underlying ovarian cancer progression can facilitate the development of effective treatment modalities. Novel diagnostic and therapeutic approaches will enable better prediction and screening of high-risk populations for ovarian cancer, providing molecular targets and directions for subsequent medical intervention. Therefore, the study and control of biological behaviors such as proliferation, invasion, and metastasis of cancer cells are necessary and have significant clinical implications for ovarian cancer treatment [12].

Chromosomal proteins are newly discovered constitutive proteins that play a crucial role in mitosis and provide attachment points for spindle fibers to pull chromosomes during cell division [2, 8, 13]. They are composed of a giant protein complex associated with DNA regions that are highly repetitive and variable between different species, and highly conserved protein parts known as centromere-associated proteins (CENPs) [23, 32]. When cells enter mitosis, CENPs assemble into kinetochores, which then attach to microtubules and drive spindle fibers to form connections between chromosomes and microtubules [26]. This allows the chromosomes to be pulled towards opposite ends of the cell, accelerating their separation and regulating the cell cycle. Recent proteomics studies have identified more than 150 members of the CENP family, which form a large protein network system divided into several subgroups based on their functions. Some CENPs, such as *CENP-A*, *CENP-E*, *CENP-H*, and *CENP-R*, are highly expressed in many cancers and may serve as potential biomarkers for malignant progression and poor prognosis [3, 14, 17, 22]. High expression levels of *CENP-O* protein have been observed in gastric cancer tissue specimens and are associated with clinical staging, tumor volume, lymph node metastasis, and shorter survival times. *CENP-O* overexpression in gastric cancer is also linked to inhibition of the ATM signaling pathway and increased expression of CCND1 and c-Jun, promoting cancer progression [5]. Similar issues related to abnormal expression and mislocalization of CENP-A and CENP-C have been reported in highly invasive malignant tumors [1, 29, 33]. Although the molecular mechanisms underlying *CENP-O* upregulation in ovarian cancer cells remain unclear, the key role of the CENP family in ovarian cancer diagnosis and treatment has gradually emerged [27]. CENPs have been shown to be potential targets for cancer therapy, with CENP-E being proposed as a potential target even before its relationship with ovarian cancer was revealed, leading to the development of chemical inhibitors for CENP-E [24]. Understanding the fundamental causes and specific mechanisms of ovarian cancer pathogenesis is crucial for its treatment, and research on the relationship between *CENP-O* and ovarian cancer may provide a new approach.

Based on the above analysis, this study detected the expression of the *CENP-O* gene in different ovarian cancer cell lines and constructed stable transfected ovarian cancer cell lines with *CENP-O* gene knockdown to investigate its effects on ovarian cancer cell proliferation and apoptosis. To further explore the molecular regulatory mechanisms underlying ovarian cancer development, this study used gene expression microarrays and bioinformatics methods to predict downstream signaling pathways that may be affected by *CENP-O*, and validated the specific molecular mechanisms of *CENP-O* gene regulation through biochemical experiments. The results showed that *CENP-O* was highly expressed in various ovarian cancer cell lines, with the highest expression level in SKOV3 cells. After shRNA lentiviral infection of SK-OV-3 cells, *CENP-O* gene expression was inhibited, with a knockdown efficiency of 79.8%. Knockdown of the *CENP-O* gene resulted in increased apoptosis and decreased cell clone numbers and proliferation in SK-OV-3 cells. In addition, endogenous knockdown of the target gene for *CENP-O* had a significant knockdown effect at the protein level in SK-OV-3 cells. Furthermore, this study analyzed the disease function analysis results using IPA and found that the target gene *CENP-O* may affect the expression of downstream genes related to tumor cell proliferation, metastasis, and apoptosis by acting on EZH2 and JUN. In summary, *CENP-O* has the potential to serve as a molecular therapeutic target, and downregulation of *CENP-O* gene expression can break the infinite proliferation ability of cancer cells and promote their apoptosis, providing a foundation and new direction for future molecular mechanism research and targeted therapy.

## Materials and methods

### Cell lines and culture conditions

Human ovarian carcinoma cell lines, ES-2, A2780, Caov-3, OVCAR-3 and SK-OV-3 were purchased from Shanghai Institute of Cell Biology, Chinese Academy of Sciences. All cell lines were cultured in Dulbecco’s Modified Eagle’s Medium (Gibco; Thermo Fisher Scientific, Inc.)All cell lines were cultured in RPMI 1640 medium containing 10% fetal bovine serum, 100 µg/mL of streptomycin, and 100 U/mL of penicillin at 37˚C in an atmosphere of 5% CO_2_. Digestion and passage were performed every day with 0.25% trypsin.

### Plasmids, lentiviral vector construction

After detecting the expression of *CENP-O* gene in human ovarian cancer cell lines using real-time quantitative PCR, SK-OV-3 and ES-2 cell lines with representative high expression levels were selected as target cells for infection. *CENP-O* RNA interference lentiviral vector (virus number: LVpGCSIL-004PSC39098-1) and control virus (virus number: psc3741) were synthesized and sequenced by Shanghai Jikai Gene Chemical Technology Co., Ltd. The cancer cells were seeded in a 6-well plate (5 × 104 cells/well) until confluence reached 60%, and then each well was infected with appropriate amounts of *CENP-O* RNA interference lentiviral vector or control virus on two culture plates according to the manufacturer’s recommendations, and named the shCENPO group and the shCtrl group, respectively. After infection, the cells were cultured in regular medium for an additional 10 h. Fluorescence intensity was observed using a fluorescence microscope (Olympus Corporation) and infection efficiency was calculated after at least 72 h of lentivirus infection. Stable cell lines were selected using corresponding antibiotics.

### Reverse transcription‑quantitative PCR (RT‑qPCR)

Total RNA was isolated using TRIzol reagent (Invitrogen; Thermo Fisher Scientific, Inc.) according to the manufacturer’s protocol. cDNA was synthesized by reverse transcription (RT) using the Promega M-MLV (Agilent Technologies, Inc.) according to the manufacturer’s protocol (Promega Corporation). The reverse transcription was carried out by incubating the reaction mixture at 73.5℃ for 7 min, followed by immediate cooling on ice-water mixture to allow annealing of the reverse transcription primers and template. The reaction mixture was then incubated at 43.5℃ for 1 h, followed by inactivation of the reverse transcriptase at 73.5℃ for 3 min. The resulting cDNA products were stored at -20℃ for later use. The cDNA product was amplified using a SYBR Green Master Mix kit (Takara Bio, Inc.) and the MX3000p (Agilent Technologies, Inc.). GAPDH was used as the internal control. The primers used were shown in Supplementary Table 1. The thermocycling conditions were as follows: 95˚C for 4 min, followed by 35 cycles of 95˚C for 15 s, 60˚C for 30 s and 72˚C for 30 s. The relative mRNA expression (CENP-O/ACTB) was determined using the 2-ΔΔCq method. The same method was used for downstream gene validation after bioinformatics analysis, and the primer information for the target genes is provided in Supplementary Table 1.

### Western blotting analysis

Proteins were extracted from cells with good growth conditions after lentiviral infection, and Western blot assay was used to detect the changes in protein expression of *CENP-O* gene after knockdown, thus determining the interference effect of the target. Cancer cells were lysed in RIPA lysis buffer (Invitrogen; Thermo Fisher Scientific, Inc.), supplemented with protease inhibitor cocktail (Roche Applied Science), and the protein concentration was measured using the BCA Protein Assay kit (Beyotime Institute of Biotechnology). The resulting protein lysates (20 µg) were subjected to 12% SDS‑PAGE separation and transferred onto PVDF membranes. After blocking with 5% non‑fat milk for 1 h at room temperature, the membranes were incubated overnight at 4℃ with primary antibodies against *CENP-O* (1:500; TA349791; OriGene Technologies, Inc.). Subsequently, the membranes were washed thrice with TBST (0.1% Tween‑20) for 5 min each time before incubation with horseradish peroxidase-conjugated secondary antibodies, including anti-mouse (1:2,000, cat. no. ZB2301, OriGene Technologies, Inc.) and anti-rabbit (1:2,000, cat. no. ZB2305, OriGene Technologies, Inc.) for 2 h at room temperature. After washing the membranes again with TBST as before, the bands were visualized using BeyoECL Plus reagent (Beyotime Institute of Biotechnology). The membranes were then stripped and subjected to re-probing with an anti-GAPDH antibody (1:5,000; cat. no. sc47724; Santa Cruz Biotechnology, Inc.) to serve as a loading control. The same method was used for downstream Western blot verification after bioinformatics analysis, and the antibody information used is provided in Supplementary Table 2. The quantification of western blotting analysis was performed with ImageJ, the gray value was quantified after the corresponding bands were selected.

### Cell proliferation assay

Cells in the logarithmic growth phase were resuspended in standard medium and seeded at a density of 2 × 10^3^ cells/well in 96-well plates. The number of EGFP-positive cells was quantified for five consecutive days using a Celigo Imaging Cytometer (Nexcelom Bioscience LLC).

### Apoptosis assay

Cells were seeded in 96-well plates at a density of 100 µl/well and cultured in a CO_2_ incubator at 37 °C for 3 days. Caspase-Glo3/7 buffer and lyophilized caspase-Glo3/7 substrate were equilibrated to room temperature. Next, 10 ml of caspase-Glo3/7 buffer was added to a brown bottle containing the substrate, and the mixture was vortexed until the substrate was completely dissolved to create caspase-Glo reaction solution. After cell counting, cell suspensions were adjusted to a concentration of 1 × 10^4^ cells/well at room temperature. A total of 100 µl/well of the respective cell groups and a negative control group with medium only were seeded in a new 96-well standard plate. The plates containing cells were shaken at 300–500 rpm for 30 min at room temperature. After incubation for 2 h, the signal intensity was measured.

### MTT assay

To validate the effect of the target gene on cell proliferation by detecting cell viability, the experimental procedure was as follows: After digestion with trypsin, cells in each group in exponential growth phase were resuspended in complete medium and counted. After shRNA lentivirus infection for 3 days, the cells were seeded into 96-well plates at a density of 2000 cells/well with 5 plates per group. The cell density of each group was observed under a microscope after the cells settled down completely. Starting from the 2nd day of seeding, 20 µL of 5 mg/mL MTT was added to each well for 4 h before the culture was stopped. After removing the medium, 100 µL of DMSO was added to dissolve formazan crystals. After 60 h of further culturing, the absorbance ratio of each group at a wavelength of 490 nm detected by an enzyme-linked immunosorbent assay (ELISA) reader was compared between the shCENPO group and the shCtrl group. Data analysis was performed using GraphPad Prism 9.5.0.

### Colony forming assay

To assess the proliferation ability of cells, their clonogenic formation capability was tested by infecting cells and seeding them onto culture plates. The experimental procedure was as follows: two groups of cells in logarithmic growth phase were digested with trypsin, resuspended in complete medium, and the cell concentration was counted after making a cell suspension. A total of 800 cells/well were seeded in each experimental group of a 6-well plate with three replicate wells. After seeding, the cells were continuously cultured in a humidified incubator for up to 12 days while changing the medium every three days. The cell status was observed regularly during the incubation period. At the end of the experiment, the cells were washed once with PBS and fixed with 1 mL of 4% paraformaldehyde per well for 30–60 min. The cells were then stained with clean and pure crystal violet staining solution (1000 µL/well) for 10–20 min and washed repeatedly with ddH_2_O. The dried cells were photographed under a fluorescence microscope (Leica Microsystems, Inc.) and the clones counted.

### PI-FACS cell cycle detection

The PI-FACS cell cycle detection was performed as previously reported [4]. In brief, when ES-2 cells reached a confluence of approximately 80%, they were digested with trypsin and suspended in complete culture medium to form a cell suspension. The collected cells were then centrifuged at 1300 rpm for 5 min in a 5 mL centrifuge tube, and the supernatant was discarded. The cell pellet was washed once with pre-cooled D-Hanks (pH = 7.2 ~ 7.4) at 4 °C. After centrifugation at 1300 rpm for 5 min, the cells were fixed with pre-cooled 75% ethanol for at least 1 h. The fixed cells were washed once with D-Hanks, then stained with a cell staining solution prepared by mixing 40×PI stock solution (2 mg/mL), 100×RNase stock solution (10 mg/mL), and 1×D-Hanks in a volume ratio of 25:10:1000. A certain volume of the staining solution (0.6-1 mL) was added according to the cell concentration to obtain a cell suspension that would achieve a flow rate of 300 ~ 800 Cell/s during detection. Finally, the cells were subjected to CytoFLEX detection and data analysis.

### β-galactosidase staining

In this study, the cell senescence β-galactosidase staining kit (BeyoTime, product manual link: http://www.beyotime.com/c0602.htm) was used to detect cellular senescence. Briefly, ES-2 cells were seeded in a 6-well plate and cultured in a CO_2_ incubator at 37 °C for 3–5 days. After removing the medium, the cells were washed with PBS once and then fixed with 1 ml of β-galactosidase fixative solution at room temperature for 15 min. Next, the fixative was removed and the cells were washed with PBS three times for 3 min each. Afterwards, 1 ml of staining working solution was added to each well and the plate was sealed with sealing film and incubated overnight at 37 °C without CO_2_ in an incubator. The total number of cells and stained cells were counted under a light microscope, and data analysis was performed using GraphPad Prism 9.5.0.

### Subcutaneous injection tumor model

All animal experiments were approved by the Ethics Board of The Second Norman Bethune Hospital of Jilin University. Four-week-old female SPF BALB/c nude mice purchased from Shanghai Lingchang Biotechnology Co. Ltd were adaptively fed for three days and subjected to tumor formation experiments without any abnormalities. An adequate amount of SK-OV-3 cells were prepared and each experimental group of logarithmically growing tumor cells was digested with trypsin and resuspended in complete medium to form a cell suspension. The cells were counted using a hemocytometer and finally resuspended in D-Hanks or PBS at a concentration of 2 × 10^7^ cells/ml, and then injected subcutaneously into the right forelimb shoulder of each animal with a disposable sterile syringe (200 ul per mouse). Based on the tumor-forming ability of the cells, tumor growth was monitored began at the time 8 days after subcutaneous injection, then tumor size and animal weight were measured for the next 20 days(two times a week). After subcutaneous injection for 28 days, or according to the actual tumor formation situation and ethical guidelines for animal welfare (tumor long and short diameter < 20 mm), the experimental animals were euthanized with an overdose of 2% pentobarbital sodium injection and confirmed to be dead by cervical dislocation. The animals were laid out on a white board and two rulers were placed on the left side and top edge for specific scale observation. Digital photographs were taken of the animals, with the tumors placed at the top for observation. The tumors were removed using surgical scissors and forceps and laid out on the white board for documentation. The weight of the tumors was measured and, according to subsequent experimental requirements, they were fixed in formaldehyde or frozen in liquid nitrogen and stored at -80 °C. Finally, the data was collated and a complete report was produced.

### Gene expression profiling and data analysis

We employed SK-OV-3 cells that were genetically modified with sh-CENP-O, alongside their respective control cells, to investigate gene expression patterns using microarray analysis. The experiment involved the extraction of total RNA from the SK-OV-3 cells and generation of biotin-modified amplified RNA (aRNA) by utilizing a GeneChip 3’IVT Express kit (Affymetrix; Thermo Fisher Scientific, Inc.), with 50–500 ng of RNA used for this purpose. Reverse transcription was initiated employing a T7 oligo (dT) primer, followed by utilization of first‑strand IVT Labeling Master Mix that yielded multiple copies of biotin-modified aRNA. The generated aRNA underwent purification and quantification before being fragmented and hybridized onto the GeneChip PrimeView™ human array (Affymetrix; Thermo Fisher Scientific, Inc.). Subsequently, we stained the chips with streptavidin-phycoerythrin (MoBiTec, Goettingen, Germany) for 10 min at 25℃ and then washed it in a GeneChip Fluidics Station 450. Microarray signals were then captured and analyzed through the use of a GeneChip Array Scanner 3000 (Thermo Fisher Scientific, Inc.). Differential gene expression was noted among our samples when exhibiting either a > 1.5-fold change or a < 0.5-fold change alongside where the P-value indicated significance (< 0.05 following correction). We leveraged Ingenuity Pathway Analysis (IPA; http://www.ingenuity.com) to conduct pathway analysis, thereby enabling us to identify significant DEGs and perform enrichment analysis.

### Statistical analysis

Each experiment was performed in triplicate. The GraphPad Prism 9.5.0 was used to perform statistical analysis of the results. Data are expressed as the mean ± standard deviation. Significant differences between two groups were analyzed using Student’s t-test. Analysis of variance analysis was used for the comparison of multiple groups and a Dunnett test was used as a post hoc test. *P* < 0.05 was used to indicate a statistically significant difference. Significance was indicated as follows: *p* ≤ 0.05 (*); *p* ≤ 0.01 (**); *p* ≤ 0.001 (***); *p* ≤ 0.0001 (****).

## Results

### *CENP-O* is highly expressed in ovarian cancer cell lines

**Fig. 1:**
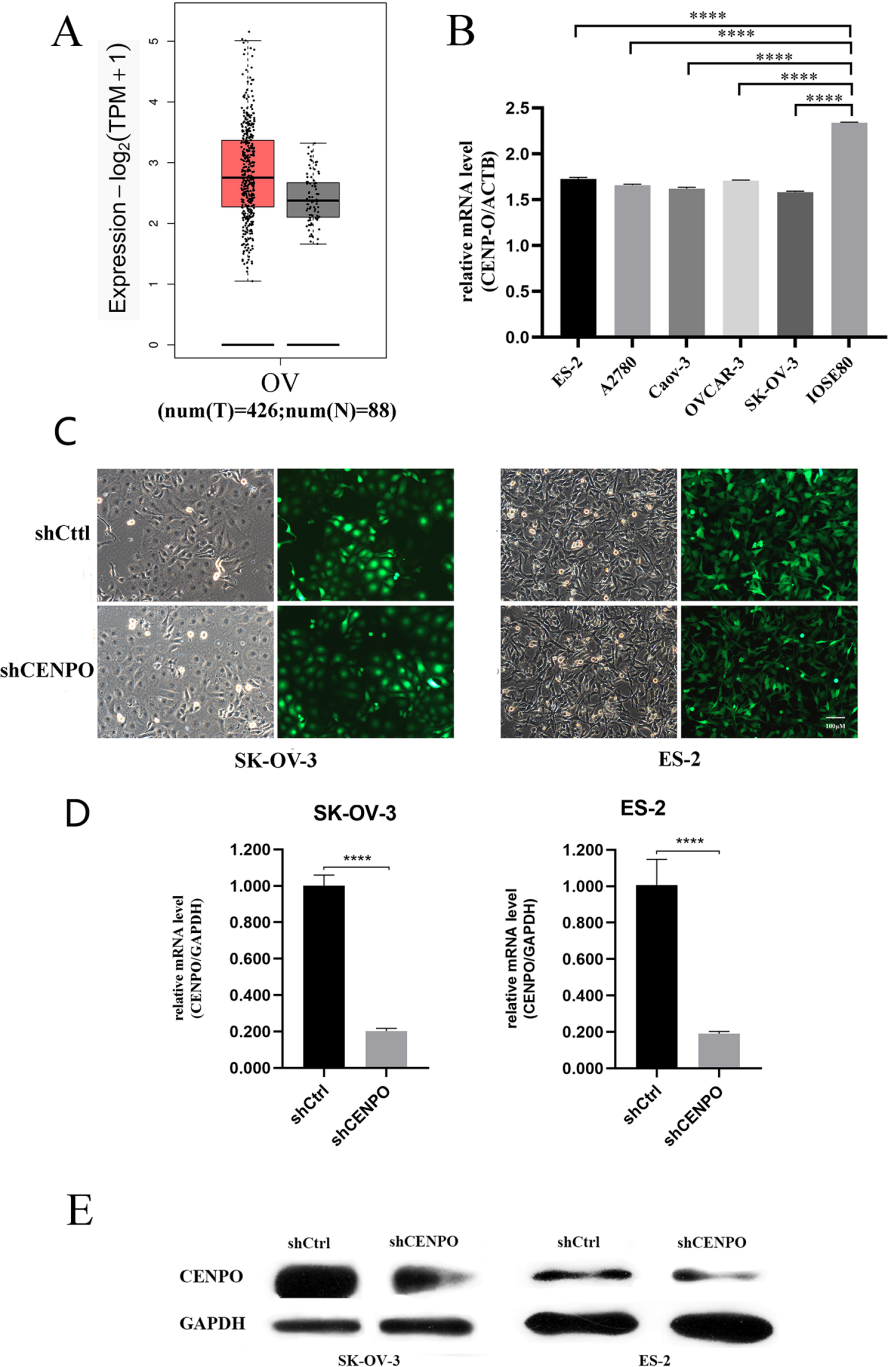
CENPO is upregulated in OV and the identification of lentivirus knockdown inefficiency. (**A**) CENPO mRNA expression levels in 426 ovarian cancer tissues and 88 normal tissues according to the TCGA and GTEx dataset (*P* < 0.0001). (**B**) mRNA expression levels in 5 ovarian cancer cell lines and 1normal ovarian cell lines. (**C**) Fluorescent image of SK-OV-3 and ES-2 cells infected with lentivirus. **D**) Comparison of mRNA expression abundance between shCtrl and shCENPO group in two cell lines. (**E**) Comparison of protein expression between shCtrl and shCENPO. Each western blot experiment was repeated three times. (Significant differences between two groups were analyzed using Student’s t-test. Significance was indicated as follows: *p* ≤ 0.05 (*); *p* ≤ 0.01 (**); *p* ≤ 0.001 (***); *p* ≤ 0.0001 (****))

It has been reported that the expression of *CENP-O* is low in normal tissues, but high in cancer tissues [11, 30]. To evaluate the significance of CENPO in ovarian cancer, the expression of CENPO in TCGA and GTEx datasets was first examined. As demonstrated in Fig. 1A, significantly increased CENPO mRNA levels were observed in cancer tissues when compared with paired normal tissues (*P* < 0.0001).To confirm these results, we used qPCR to examine the expression levels of *CENP-O* across five different ovarian cancer cell lines against normal ovarian cell lines IOSE80. As indicated in Fig. 1B, the results revealed that *CENP-O* gene expression had a ΔCt value of 8.97 in ES-2 cells, 7.95 in A2780 cells, 8.31 in CaOV3 cells, 9.12 in OVCAR-3 cells and 8.09 in SK-OV-3 cells. The ΔCt values across all five cell lines were less than 12, indicating that expression levels of *CENP-O* were high among these cell lines. Among the cell lines, A2780 exhibited the highest level of *CENP-O* gene expression while OVCAR-3 demonstrated the lowest. To proceed with subsequent experiments, we selected representative human ovarian cancer cell lines, SK-OV-3 and ES-2, for lentiviral infection experiments.

### *CENP-O* regulates ovarian carcinoma cell growth and apoptosis in vitro

**Fig. 2:**
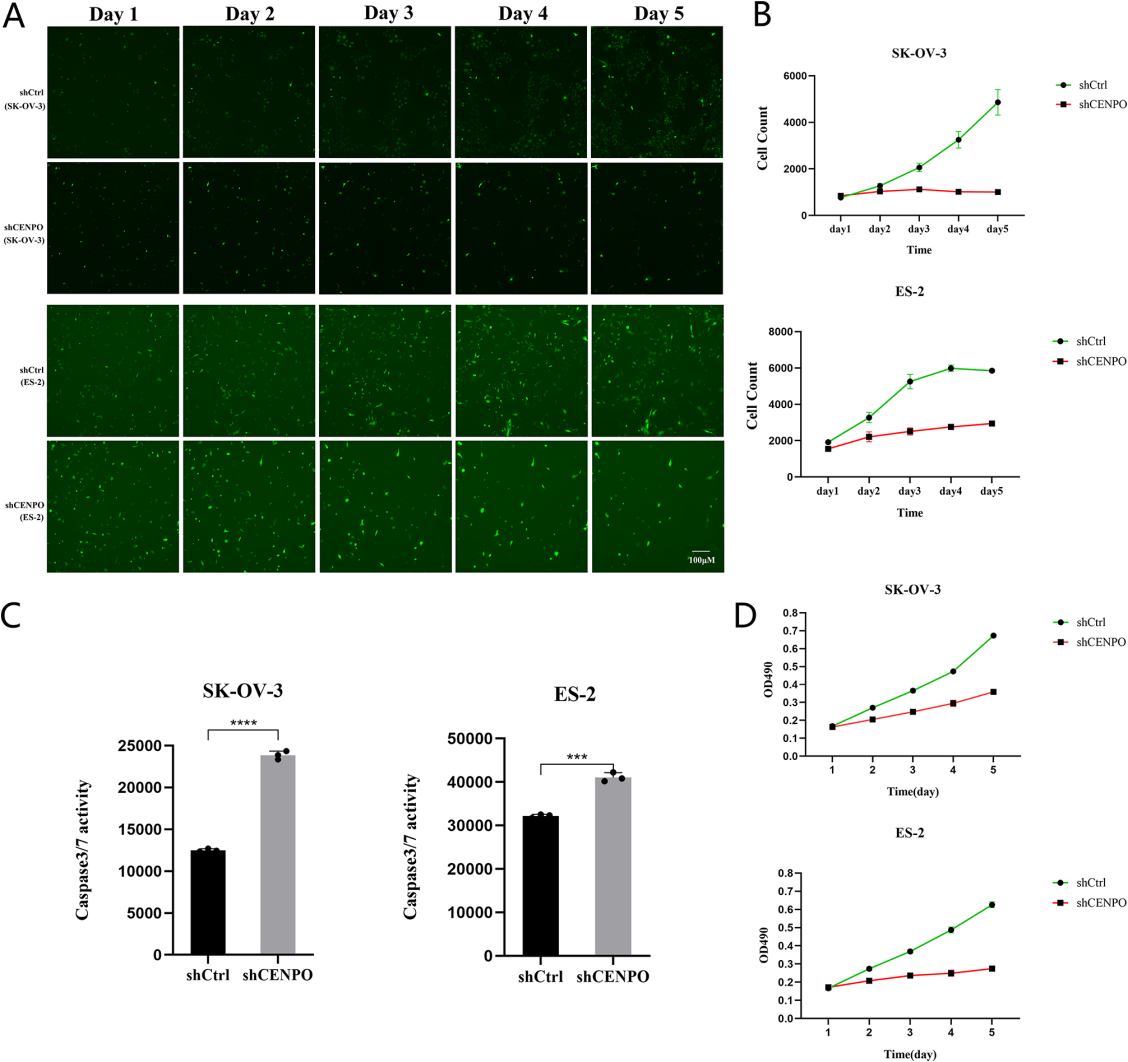
Functional role of CENP-O in regulating ovarian cancer cell growth and apoptosis in vitro. (**A**) Fluorescent image of SK-OV-3 and ES-2 cells migration area. (**B**) The proliferation rate/multiplication factor of shCtrl and shCENPO group of two cell lines determined by Celigo. (**C**) Caspase3/7 activity and (**D**) MTT assays were performed to determine the proliferation of ovarian cancer cells transduced with the CENP-O shRNA lentivirus ( doubling time of SK-OV-3: 20–48 h, ES-2: 28–36 h). (Significant differences between two groups were analyzed using Student’s t-test. Significance was indicated as follows: *p* ≤ 0.05 (*); *p* ≤ 0.01 (**); *p* ≤ 0.001 (***); *p* ≤ 0.0001 (****))

To investigate the biological effects of *CENP-O* in ovarian cancer, we conducted lentivirus knockdown experiments in SK-OV-3 and ES-2 cell lines. Lentivirus containing RNA interference sequences targeting *CENP-O* gene were used to infect SK-OV-3 cells, and cell status and infection efficiency were observed. Cell status was good without significant death, and the shCENPO group’s cell status was similar to that of the shCtrl group. Fluorescence microscopy as shown in Fig. 1C revealed that the infection efficiency exceeded 80%, with the rate of infection greater than 50% and normal cell status. After transfection with *CENP-O* shRNA, mRNA and protein levels were significantly reduced compared to the control group (*P* < 0.05; Fig. 1D and E). Celigo and caspase3/7 activity and MTT assay were used to study the effect of *CENP-O* on cell growth. Celigo results showed that after continuous detection for 5 days, there was no significant change in the number of cells in the experimental group, and the cell proliferation rate was significantly inhibited (Fig. 2A, B). Caspase3/7 activity results showed a significant increase in apoptosis of SK-OV-3 cells in the experimental group, suggesting a significant correlation between the *CENP-O* gene and apoptosis in SK-OV-3 cells (Fig. 2C). MTT results displayed the change in the absorbance per unit time at a wavelength of 490 nm by enzyme-labeling instrument of shCENPO group and shCtrl group varied over time as shown in Fig. 2D. Compared with the shCtrl group, the cell proliferation of the shCENPO group was slowed down, and after continuous detection for 5 days, the proliferation rate of SK-OV-3 cells in the experimental group was significantly inhibited. These results indicate that reducing the expression of *CENP-O* may inhibit cell growth and promote apoptosis, suggesting a significant correlation between the *CENP-O* gene and the proliferation ability of SK-OV-3 and ES-2 cells.

### *CENP-O* regulates ovarian carcinoma cell growth cycle and cellular activity in vitro

**Fig. 3:**
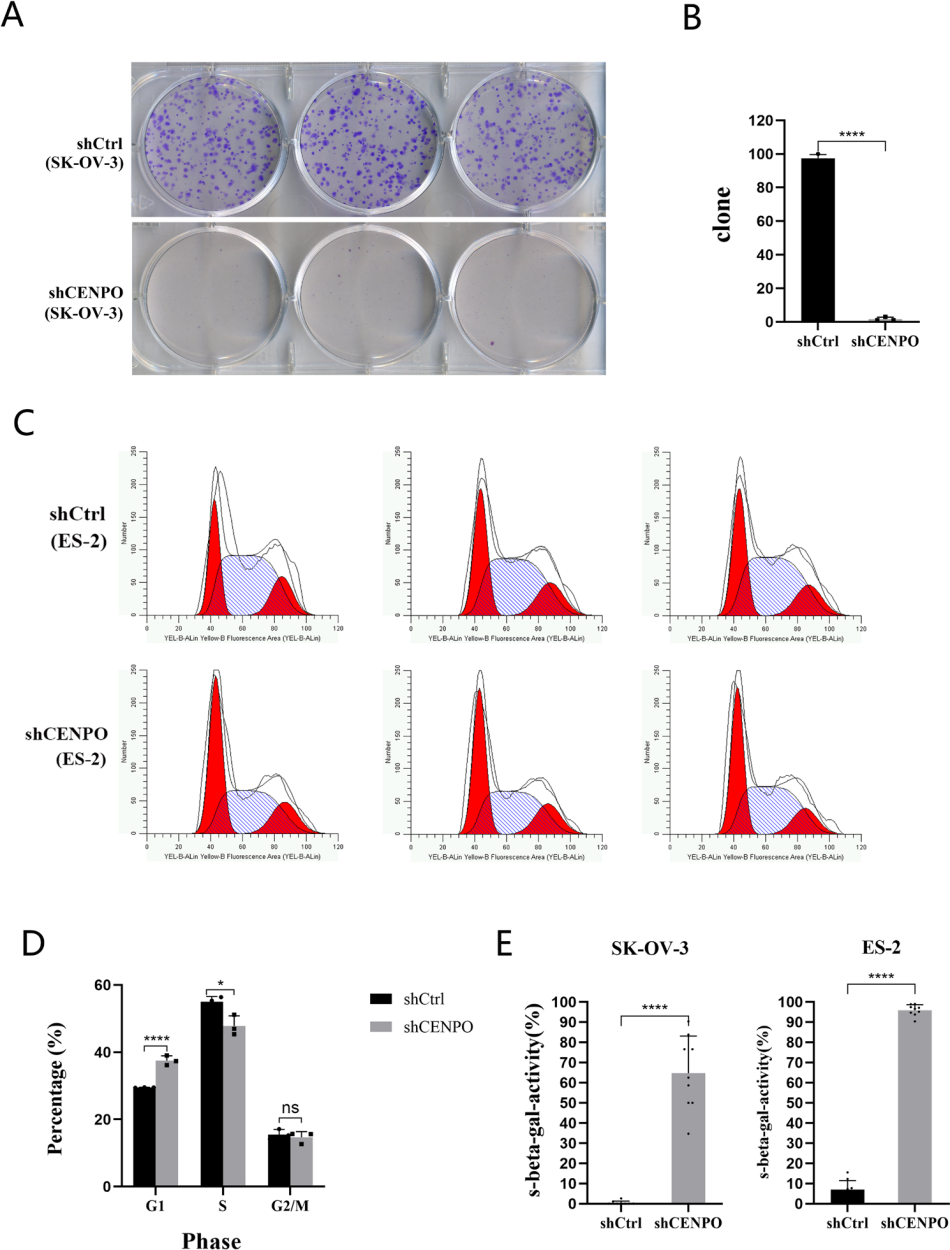
Functional role of CENP-O in regulating ovarian cancer cell cycle and clonal formation in vitro. (**A**) A colony formation assay was performed using SK-OV-3 cells transduced with the CENPO shRNA lentivirus. (**B**) Number of SK-OV-3 cell colonies that formed following transduction with the CENPO shRNA lentivirus. (**C**) Flow cytometry was used to analyze cell populations of different stages of the cell cycle. (**D**) The proportion of ES-2 cells in different cell cycles. (**E**) β-galactosidase activity assay was performed using SK-OV-3 and ES-2 cells transduced with the CENPO shRNA lentivirus. (Significant differences between two groups were analyzed using Student’s t-test. Significance was indicated as follows: *p* ≤ 0.05 (*); *p* ≤ 0.01 (**); *p* ≤ 0.001 (***); *p* ≤ 0.0001 (****))

To further investigate the effects of *CENP-O* on ovarian cancer cell growth cycle and cell activity, we conducted a clonogenic assay, PI-FACS cell cycle analysis, and β-galactosidase staining assay. Clonogenic assay is an effective method to measure cell proliferation ability. In this study, we examined the cloning ability of cells in the shCENPO group and shCtrl group on cell culture plates to indicate cell proliferation ability. As shown in Fig. 3A and B, the number of SK-OV-3 cell clusters in the shCENPO group decreased significantly (*p* < 0.01), suggesting that downregulation of *CENP-O* expression inhibited colony formation of GC cells, consistent with the previous observation of cell proliferation. Next, we verified the effect of the target gene on the cell growth cycle by detecting the amount of DNA in the cells. During cell cycle analysis, PI fluorescence intensity was detected by flow cytometry to directly reflect the DNA distribution status of ES-2 cells at various phase of cell cycle, and the percentage of each phase was calculated, as shown in Fig. 3C. Quantitative analysis revealed that compared with the control group, the number of cells in G1 phase increased (*P* < 0.05) and the number of cells in S phase decreased (*P* < 0.05) in the shCENPO group, while the number of cells in G2/M phase showed no significant change (Fig. 3D), suggesting that cell division was arrested in G1 phase and cell growth was inhibited. Subsequently, we verified the effect of the target gene on cell proliferation by detecting cellular senescence. Most normal cells enter senescence after a limited number of divisions and cannot divide under some conventional stimuli. Their cell cycle, genes, and protein expression all change. Senescent cells have larger volume and express high enzyme activity of β-galactosidase at pH 6.0. The results showed that the β-galactosidase activity in both cell lines increased significantly compared to the control group (Fig. 3E), indicating that cell proliferation was inhibited. In conclusion, the down-regulation of *CENP-O* leads to the cessation of ovarian cancer cell growth and abnormal division, which may be closely related to the biological effects of ovarian cancer.

### Downregulation of *CENP-O* reduces tumorigenicity in SK-OV-3 subcutaneous injection nude mice

**Fig. 4:**
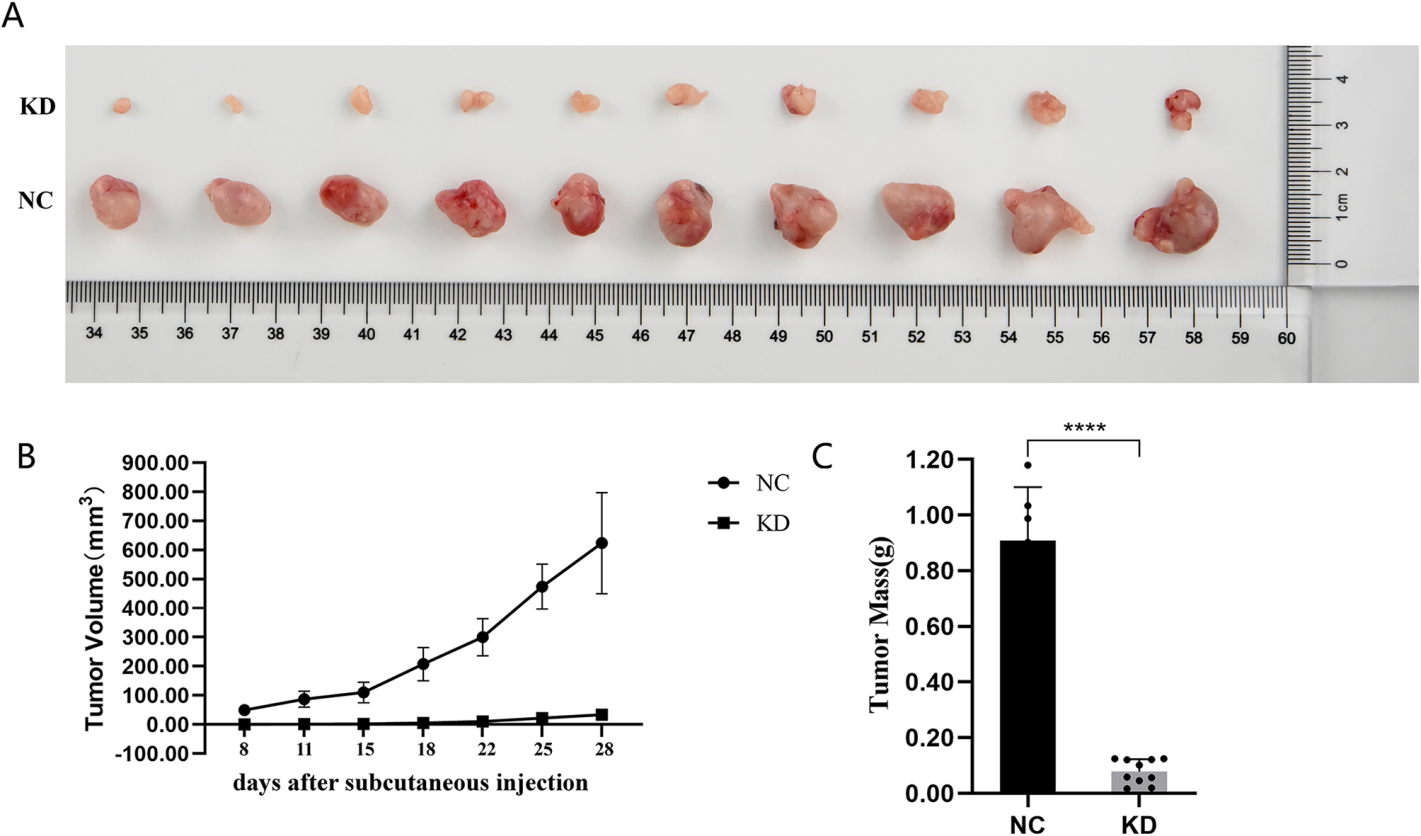
Downregulation of CENP-O expression reduces tumorigenicity in SK-OV-3 subcutaneous injection nude mice(*n* = 3). (**A, B**) Tumor volumes between the control and the CENP-O shRNA groups were compared. (**C**) Tumor weights between the control and the CENP-O shRNA groups were compared on the day 26 after the subcutaneous injection of SK-OV-3. (Significant differences between two groups were analyzed using Student’s t-test. Significance was indicated as follows: *p* ≤ 0.05 (*); *p* ≤ 0.01 (**); *p* ≤ 0.001 (***); *p* ≤ 0.0001 (****))

In this study, we used BALB/c nude mice to conduct an ovarian cancer tumorigenesis experiment to investigate the effect of *CENP-O* on ovarian cancer biological effects in vivo. As shown in Fig. 4A, after injecting SK-OV-3 cells transfected with *CENP-O* shRNA, the tumors in mice did not increase significantly over time, while the tumors in the control group increased visibly with time. The weight of the formed tumors was measured, as shown in Fig. 4B. The weight of the tumors in the control group increased significantly over time, while the weight of the tumors in the experimental group remained at a low level. On the 26th day of tumorigenesis, the weight of the tumors was measured, and the results shown in Fig. 4C indicated that the weight of the shCENPO group tumors was significantly lower than that of the control group (*P* < 0.05), suggesting that tumors formed by the experimental mice had lower proliferation ability compared to those formed by the control cells. Taken together, *CENP-O* may participate in the proliferation of ovarian cancer cells in vivo.

### CENP-O silencing is involved in a variety of cellular activity related signaling pathways

**Fig. 5:**
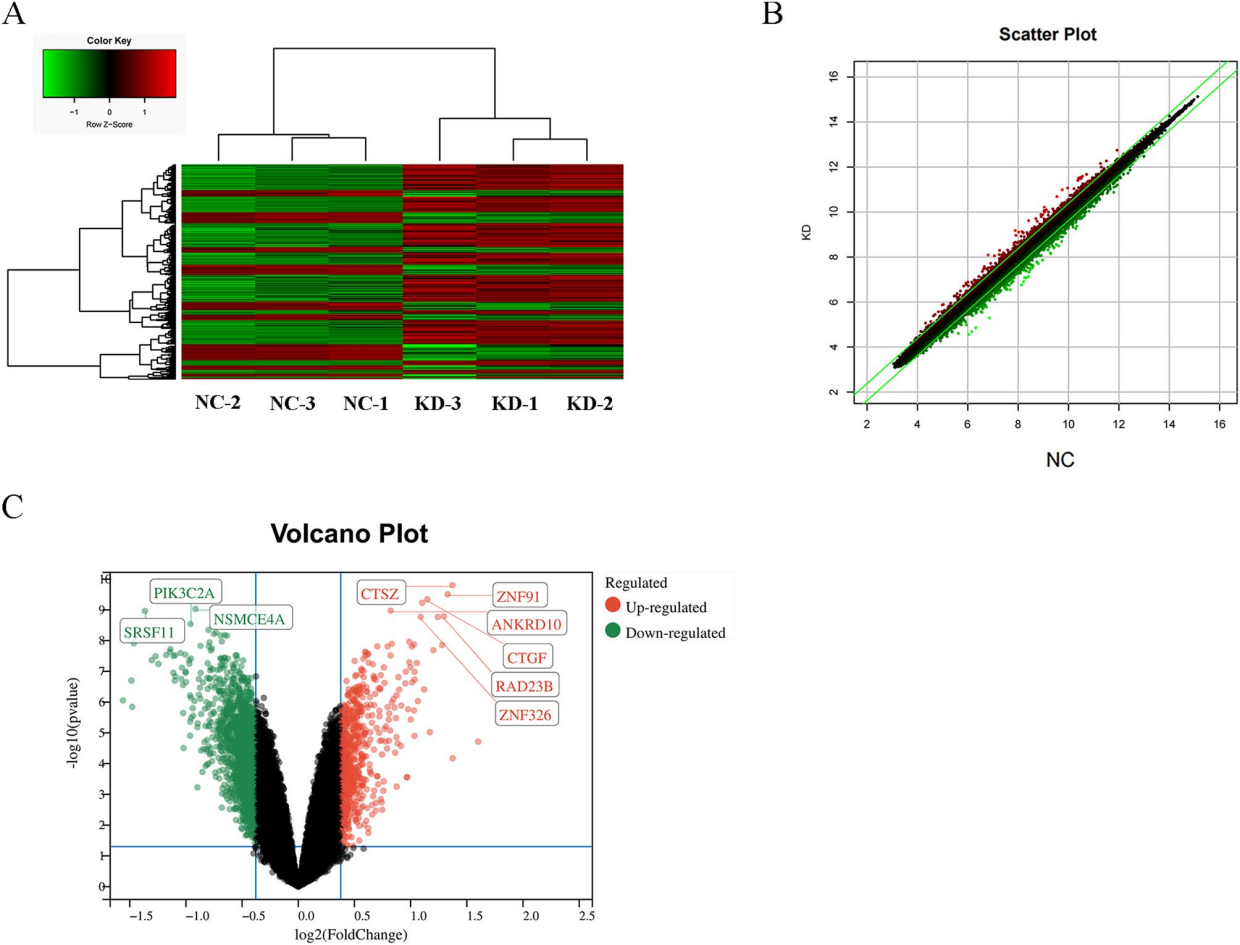
Analysis of significant difference between shCtrl group and shCENPO group after transfection of SK-OV-3 with CENP-O shRNA lentivirus. (**A**) Hierarchical clustering assay of the control and the CENP-O shRNA groups were performed, Red/green indicates a relatively upregulated/downregulated expression level of genes, black indicates that there is no significant change in gene expression, and gray indicates that the gene signal intensity was not detected. (**B**) Scatter plot of the control and the CENP-O shRNA groups drawn based on the significant difference analysis. The red dots outside the interval represent probe sets that are relatively upregulated in the KD group, while the green dots represent probe sets that are relatively upregulated in the NC group. (**C**) Volcano Plot of the control and the CENP-O shRNA groups drawed based on the significant difference analysis. The red dots represent differentially expressed genes that were screened based on the criteria of |Fold Change|≥1.3 and FDR < 0.05, while the gray dots represent other genes with no significant differences in expression

**Fig. 6:**
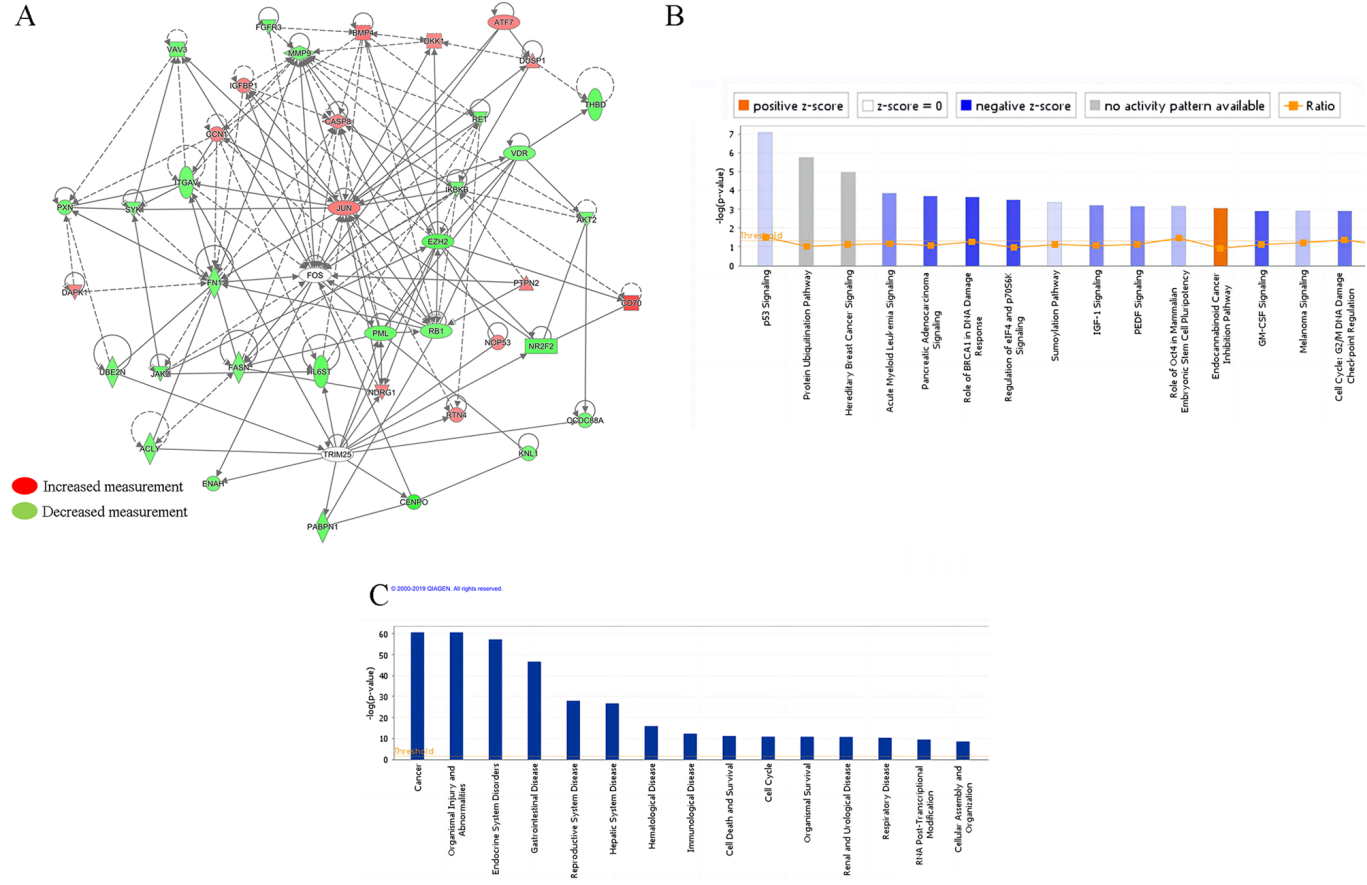
CENP-O is involved in a variety of cellular activity related signaling pathways. (**A**) Molecular interaction network analysis based on IPA. (**B, C**) CENPO may be involved in different biological processes, molecular functions and diseases according to IPA analysis. (**B**) Classical path enrichment analysis statistics. (**C**) Statistical analysis of disease and functional enrichment

To investigate the molecular mechanisms underlying *CENP-O* mediated regulation of ovarian cancer cell proliferation, we utilized a microarray platform to compare gene expression profiles between SK-OV-3 cells with knocked down *CENP-O* and control cells. We first conducted significance analysis using a linear model based on empirical Bayesian distribution to calculate P-values for significant differences and corrected the significant difference levels (FDR) using the Benjamini-Hochberg method. The screening criteria for significantly different genes were |Fold Change|≥1.3 and FDR < 0.05. Figure 5A shows a heatmap of the expression profiles of differentially expressed genes identified using the above screening criteria for SK-OV-3 cells with knocked down *CENP-O* and control cells samples by hierarchical clustering. Red indicates relatively upregulated expression levels, green indicates relatively downregulated expression levels, black indicates no significant changes in gene expression, and gray indicates signal strength not detected. In addition to significance difference level analysis, we also drawed a scatter plot (Fig. 5B) to show the distribution of signal intensities between experimental and control groups on a Cartesian coordinate plane. Moreover, a volcano plot was generated by combining fold change and significance testing results to display the significant differences between the two sample groups (Fig. 5C). This analysis identified 1045 downregulated genes and 523 upregulated genes. Biological information analysis was performed on the differentially expressed genes obtained, and the disease function analysis results of the interaction network analysis based on IPA were used to identify genes associated with tumor cell proliferation, migration, and apoptosis. Genes that were selected to inhibit tumor cell proliferation or promote tumor cell apoptosis were used to drew a gene relationship network map. Results showed that the target gene *CENP-O* may affect downstream gene expression by acting on EZH2 and JUN, thus affecting cell proliferation, migration, and apoptosis (Fig. 6A). The IPA classic pathway analysis displayed that the Pancreatic Adenocarcinoma Signaling was significantly inhibited (Z-score=-2.524) (Fig. 6B). In addition, the IPA disease and function analysis showed that cancer, organismal injury or abnormalities, endocrine system disorders, and gastrointestinal diseases were closely related to *CENP-O* dysregulation (Fig. 6C). The disease and function heatmap displayed how changes in differentially expressed gene expression levels affected the activation and inhibition of diseases and functions.

**Fig. 7:**
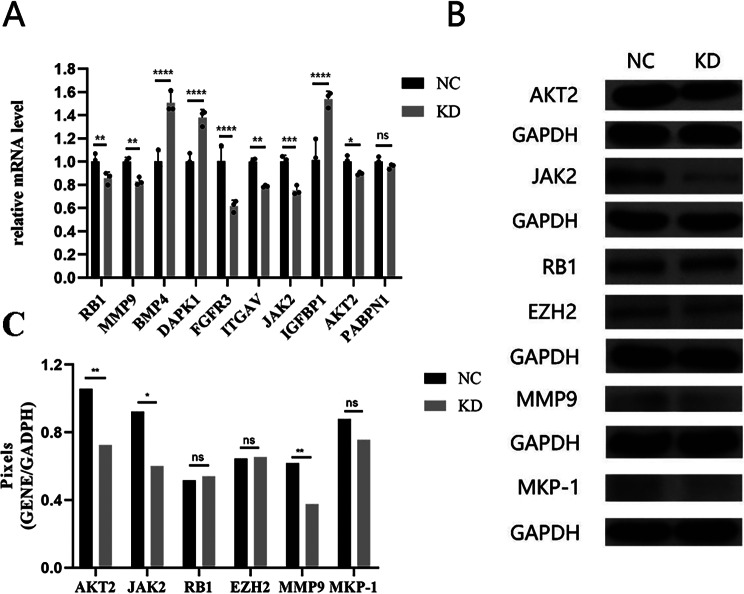
Selection and validation of downstream genes. (**A**) RB1, MMP9, BMP4, DAPK1, FGFR3, ITGAV, JAK2, IGFBP1, AKT2, PABPN1 were selected based on IPA analysis and verified using qPCR. (**B**) JAK2, EZH2, RB1, AKT2, MMP9, and MKP-1 were chosen for Western-blot validation. (**C**) The gray analysis results in Figure B. (Significant differences between two groups were analyzed using Student’s t-test. Significance was indicated as follows: *p* ≤ 0.05 (*); *p* ≤ 0.01 (**); *p* ≤ 0.001 (***); *p* ≤ 0.0001 (****))

Based on the bioinformatics analyses above, we speculate that the target gene *CENP-O* may play a role in ovarian cancer by regulating the expression of RB1, MMP9, BMP4, DAPK1, FGFR3, ITGAV, JAK2, IGFBP1, AKT2, PABPN1. These genes are associated with a variety of signaling pathways, including cell proliferation, apoptosis, differentiation, and intercellular communication. We verified this speculation using qPCR and selected the genes JAK2, EZH2, RB1, AKT2, MMP9, and MKP-1 for Western-blot validation. The qPCR results showed that except for PABPN1, all other genes were significantly different from the control group, suggesting that the expression of differentially expressed genes may play a functional role in ovarian cancer (Fig. 7A). Western-blot results showed that AKT2, JAK2, and MMP9 protein expression were significantly downregulated(Fig. 7B), which was consistent with the qPCR results. Based on the results of the experiments and bioinformatics analyses, it is inferred that the target gene *CENP-O* exerts its effects in ovarian cancer cell disease by regulating genes such as AKT2, JAK2, and MMP9 in *SK-OV-3* cells.

## Discussion

As a member of the CENP family, *CENP-O* plays a unique role in spindle damage recovery and is involved in chromatin remodeling and heterochromatin formation [26]. There are over 40 CENP proteins that have been identified and abnormalities in their localization or overexpression can lead to cell division disorder and chromosomal aneuploidy, which are closely related to cancer [10, 21]. Previous studies have shown that many malignant tumors exhibit high expression of *CENP-H* or *CENP-F* [16, 19]. Although there is increasing research on the CENP family and its close relationship with different types of cancers, relatively little research has been done on the relationship between *CENP-O* and tumors, especially ovarian cancer. In this study, the expression of *CENP-O* in ovarian cancer and its effect on the biological behavior of ovarian cancer cells were detected to investigate the significance of *CENP-O* in the diagnosis and treatment of ovarian epithelial cancer.

In order to investigate the universality of high expression of the *CENP-O* gene in ovarian cancer cell lines, we employed a wide range of ovarian cancer cell lines. Ovarian cancer cell lines ES-2, A2780, Caov-3, OVCAR-3, and SK-OV-3 were selected as the primary research objects. The ES-2 cell line originates from a surgical tumor specimen of a 47-year-old African American woman with ovarian clear cell carcinoma. The tumor is a poorly differentiated ovarian clear cell carcinoma that forms tumors in mice. These cells exhibit low to moderate tolerance to various chemotherapy drugs, including doxorubicin, cisplatin, carmustine, etoposide, and cyanomorpholinodoxorubicin (MRA-CN).The A2780 human ovarian cancer cell line is derived from tumor tissue of an untreated patient. The cells grow as a monolayer and are suspended in a spinning culture vessel. A2780 is the parental strain of A2780 cisplatin-resistant cell line (A2780 CP) and A2780 adriamycin-resistant cell line (A2780 ADR).Caov-3 cells were established in 1976 from the ovarian adenocarcinoma tissue of a 54-year-old Caucasian woman. OVCAR-3 cells were established by T.C. Hamilton in 1982 from malignant ascites of a patient with progressive ovarian adenocarcinoma. SK-OV-3 cells were isolated by G. Trempe and L.J. Old in 1973 from the ascites of a patient with ovarian tumor. SK-OV-3 cells demonstrate resistance to tumor necrosis factor (TNF) and several cytotoxic agents including bleomycin, cisplatin, and adriamycin. The expression levels of *CENP-O* gene in the five cell lines were tested by qPCR, and the expression levels were high in all five cell lines, with A2780 showing the highest expression level. The high expression of *CENP-O* in the five cell lines was consistent with the results of data mining from TCGA and GEPIA analysis. In 2019, Yi Cao et al. [5]first reported a significant increase in *CENP-O* mRNA levels in cancer tissues. Suppressing *CENP-O* expression could reduce gastric cancer cell proliferation, while overexpression of *CENP-O* promoted gastric cancer tumor growth, which was further confirmed in subcutaneous xenograft tumor models. Additionally, microarray analysis suggested that *CENP-O* may be an important regulatory factor in cancer cell death, angiogenesis, cell proliferation, apoptosis, and gastric cancer development.

ES-2 had high expression in the RT-qPCR compared with other cell lines and SK-OV-3 are tolerant to tumor necrosis factor and several cytotoxic drugs including diphtheria toxin, cisplatin, and doxorubicin so that they could better simulate the clinical situation. Therefore, these two cell lines were selected for further experimental verification. The biological functional changes between the gene knockdown group and the control group were compared by knocking down *CENP-O* gene expression in SK-OV-3 and ES-2 cells as much as possible. After shRNA lentivirus infection, the mRNA expression level of *CENP-O* gene was significantly inhibited in SK-OV-3 cells, and knockdown was effective for subsequent research. This study demonstrated that after *CENP-O* gene knockdown, the SK-OV-3 cell clone formation ability decreased, the cell cycle was arrested in the G1 phase, proliferation decreased, cell doubling time was increased, and protein expression was significantly reduced. This supported that the *CENP-O* gene played a role in promoting ovarian cancer cell proliferation and increasing its cancer stem cell-like features, acting as an oncogene. In studying the effects of *CENP-O* on ovarian cancer cell apoptosis, caspase 3/7 detection results suggested an increase in apoptotic cells in SK-OV-3 and ES-2 cells after *CENP-O* gene knockdown. These research results were also confirmed in in vivo experiments: after injecting SK-OV-3 cells transfected with *CENP-O* shRNA, there was no significant increase in tumor size over time in mice, while the tumor volume and weight of the control group increased macroscopically over time. This result suggests that *CENP-O* may be involved in the proliferative effect of ovarian cancer cells in vivo.

In recent years, investigations into the role of the centromere protein O (*CENP-O*) in cancer pathogenesis have garnered substantial attention. Previous studies have hinted at *CENP-O*’s involvement in cancer proliferation and apoptosis evasion, particularly notable in gastric cancer cells [5]. This underscores its potential as a pivotal player in cancer progression. Moreover, emerging evidence positions *CENP-O* as a promising biomarker for cancer diagnosis and a prospective target for therapeutic intervention [27]. A pivotal discovery highlighted the overexpression of *CENP-E* in serous ovarian cancer, underscoring its relevance in tumorigenesis and bolstering the rationale for targeting CENP proteins in cancer therapy [9]. Against this backdrop, our investigation delves into the intricate mechanisms governing *CENP-O*’s activity in ovarian cancer cells, shedding light on its downstream effects on gene expression, notably implicating EZH2 and JUN signaling pathways. Intriguingly, dysregulation of *CENP-O* correlates closely with a spectrum of diseases, encompassing cancer, endocrine disorders, and gastrointestinal ailments, unveiling its multifaceted role in disease pathophysiology.

Building upon these findings, our study demonstrates the pivotal role of *CENP-O* in promoting ovarian cancer cell clonogenicity while suppressing apoptosis, mirroring findings in various cancer contexts. This underscores the significance of CENP proteins in driving oncogenesis and underscores their potential as therapeutic targets. However, the journey towards clinical translation necessitates rigorous basic and clinical investigations to delineate *CENP-O*’s precise role in ovarian cancer progression. While our study lays the groundwork for understanding *CENP-O*’s implication in ovarian cancer, several avenues warrant further exploration. Key among these is deciphering its impact on tumor metastasis and elucidating the underlying molecular mechanisms driving its oncogenic functions. Moreover, bridging the gap between bench and bedside necessitates robust clinical validation to harness *CENP-O*’s potential for improving diagnostic accuracy and treatment efficacy in ovarian cancer. In conclusion, our study unveils a novel dimension to ovarian cancer pathogenesis through deciphering the role of *CENP-O*. While the road ahead may be fraught with challenges, the promise of leveraging *CENP-O* as a diagnostic biomarker and therapeutic target offers renewed hope in the fight against ovarian cancer. As we navigate this uncharted territory, continued exploration of *CENP-O*’s intricacies promises to yield invaluable insights, paving the way for enhanced diagnostic modalities and novel therapeutic avenues in ovarian cancer management.

### Electronic supplementary material

Below is the link to the electronic supplementary material.

Supplementary Material 1

## Data Availability

No datasets were generated or analysed during the current study.
