# Supplementary material for: High expression levels of centromere protein O participates in cell proliferation of human ovarian cancer
Source: J Ovarian Res. 2024 Jun 18;17:126. doi: 10.1186/s13048-024-01452-x (PMC11184697; doi:10.1186/s13048-024-01452-x)

## Supplementary Materials

Supplementary table 1: the primer information for the target genes

| **Gene Name** | **Forward** | **Reverse** |
| --- | --- | --- |
| GAPDH | TGACTTCAACAGCGACACCCA | CACCCTGTTGCTGTAGCCAAA |
| RB1 | CACACACTCCAGTTAGGACTGT | CAAGTTTGTATCGCTGTGATCCA |
| MMP9 | GCTGGGCTTAGATCATTCCT | CATTCACGTCGTCCTTATGC |
| BMP4 | CCTGGGCACCTCATCACA | CATAGTTTGGCTGCTTCTC |
| DAPK1 | AATGGTGTTTACTACCTGCACTC | CTCAGGAGCGACAAACTCTGG |
| FGFR3 | CCCAAATGGGAGCTGTCTCG | CCCGGTCCTTGTCAATGCC |
| ITGAV | CATTCCTATTCTCTGAAGTCGTC | ACAGCCAGTAGCAACAATCC |
| JAK2 | ATCCACCCAACCATGTCTTCC | ATTCCATGCCGATAGGCTCTG |
| IGFBP1 | TCAGTACCTATGATGGCTCG | TATCTGGCAGTTGGGGTC |
| AKT2 | GCGGAAGGAAGTCATCATTG | GTGGGTCTGGAAGGCATAC |
| PABPN1 | TCTATGTTGGCAATGTGGAC | CCTTGATTTGCCTTCCTCT |

Supplementary table 2: the antibody information for the target genes

| **Antibody** | **Species** | **Dilution ratio** | **Molecular weight** |
| --- | --- | --- | --- |
| JAK2 | Rabbit | 1:1000 | 125 kDa |
| EZH2 | Mouse | 1:500 | 86 kDa |
| RB1 | Mouse | 1:500 | 110 kDa |
| AKT2 | Mouse | 1:1000 | 55 kDa |
| MMP9 | Rabbit | 1:1000 | 84, 92 kDa |
| MKP-1 | Rabbit | 1:500 | 39 kDa |
| GAPDH | Mouse | 1:1000 | 36 kDa |
| Rabbit IgG | Rabbit | 1:2000 |  |
| Mouse IgG | Mouse | 1:2000 |  |

Supplementary figures:

Figure 1-7: Raw data from Figures 1 and 7

Figure 1: Raw data of FIG 1 SK-OV-3


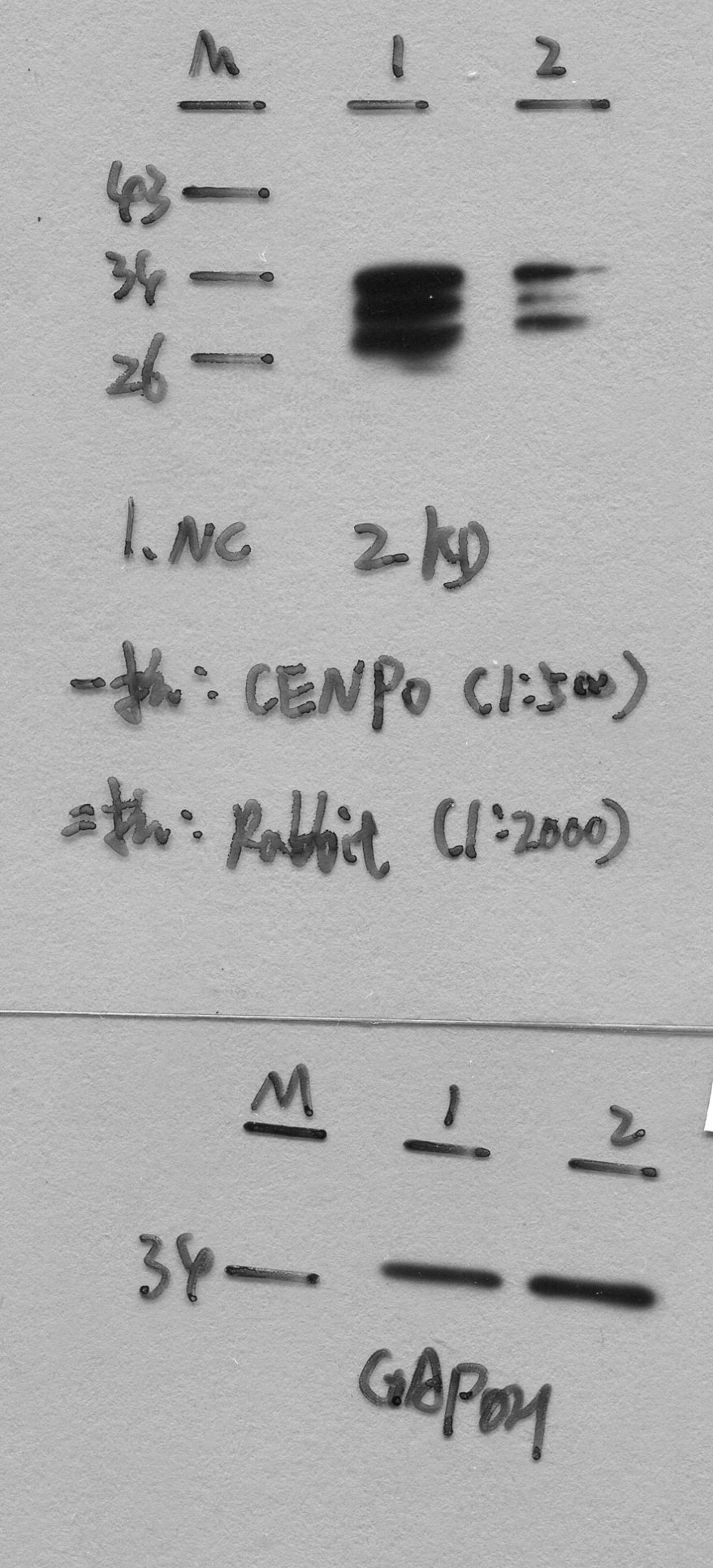


Figure 2: Raw data of FIG1 ES-2


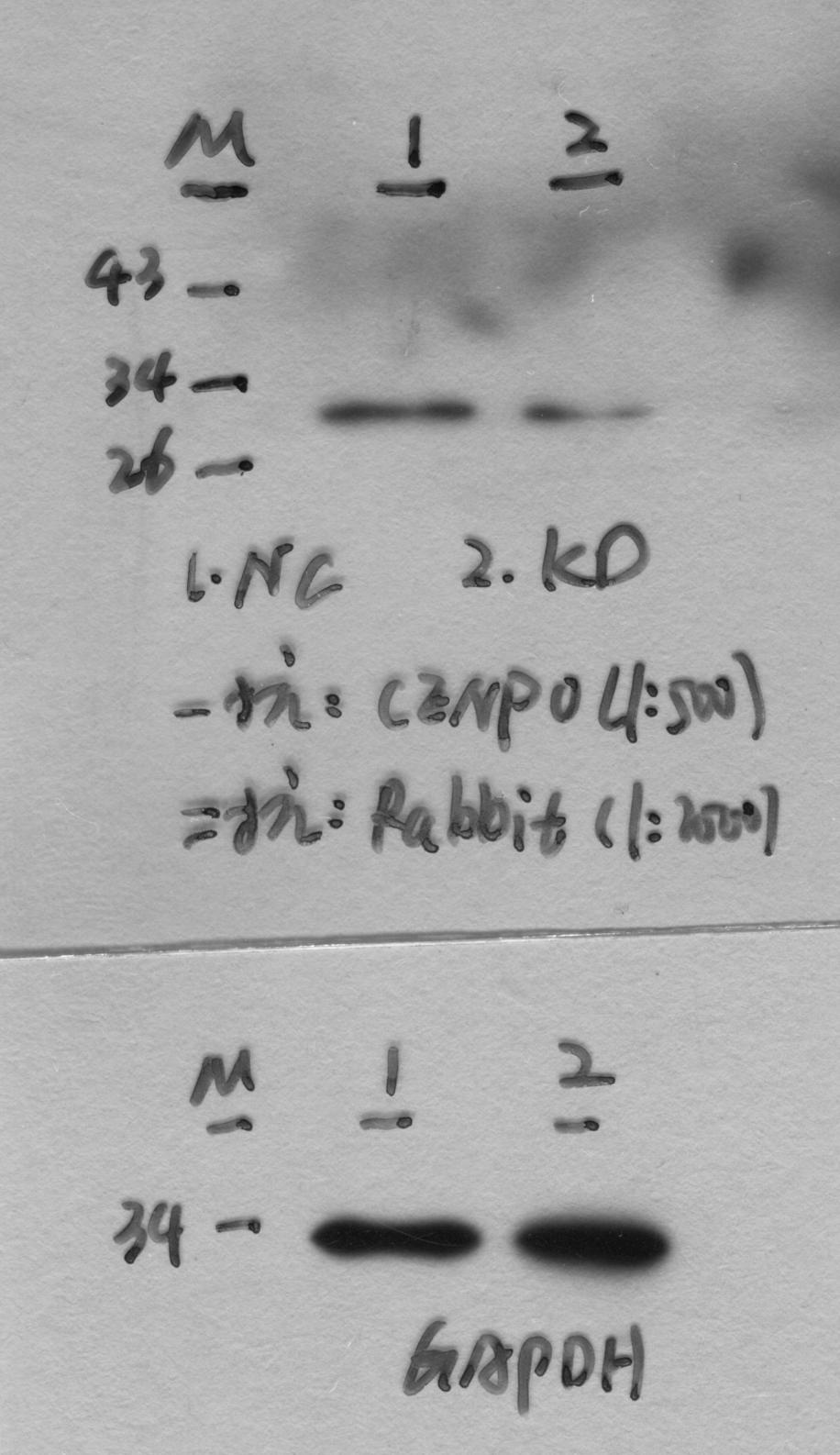


Figure 3: Raw data of FIG 7 RB1


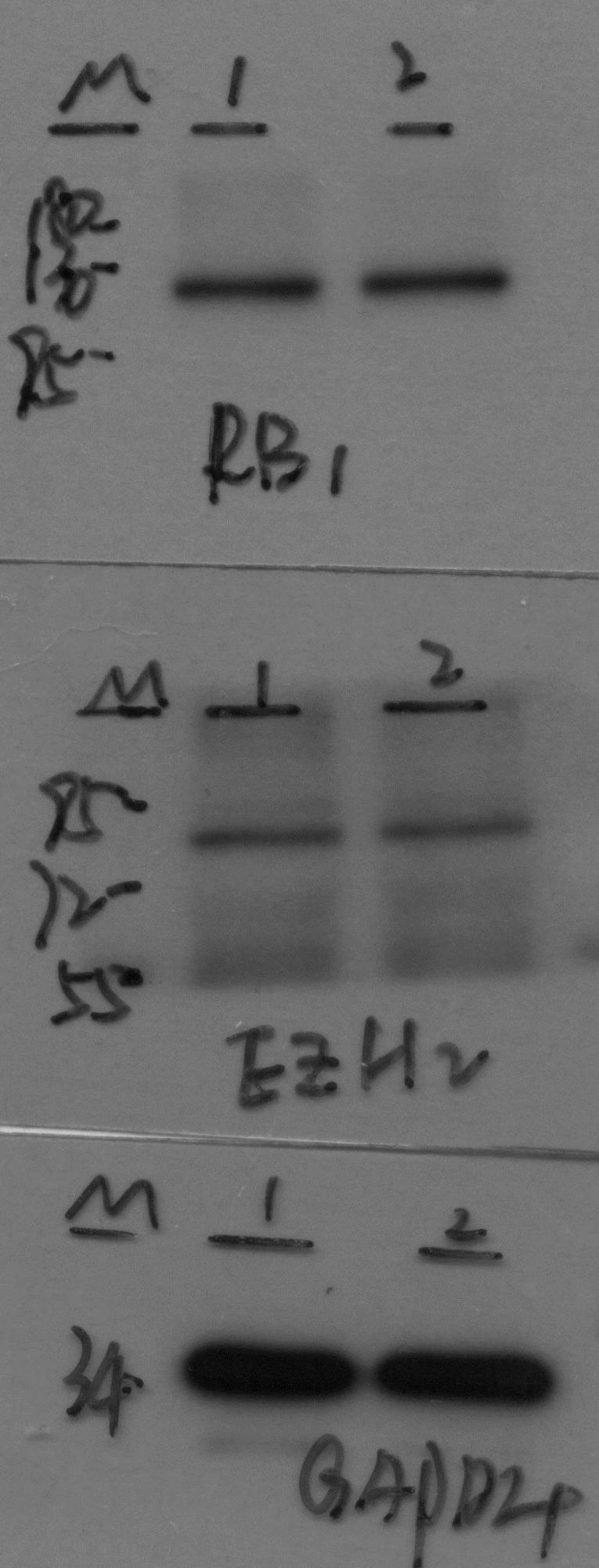


Figure 4: Raw data of FIG 7 MMP9


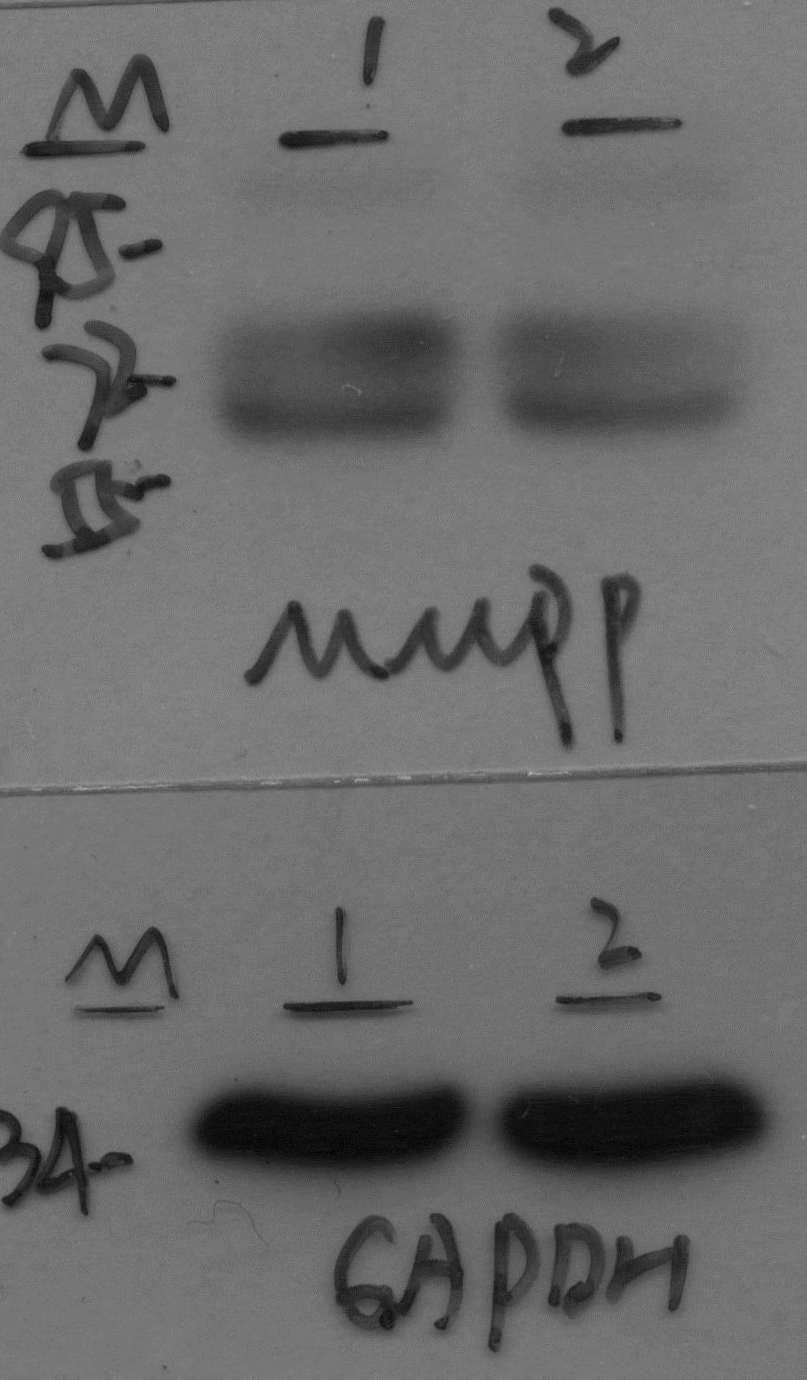


Figure 5: Raw data of FIG 7 MRP-1


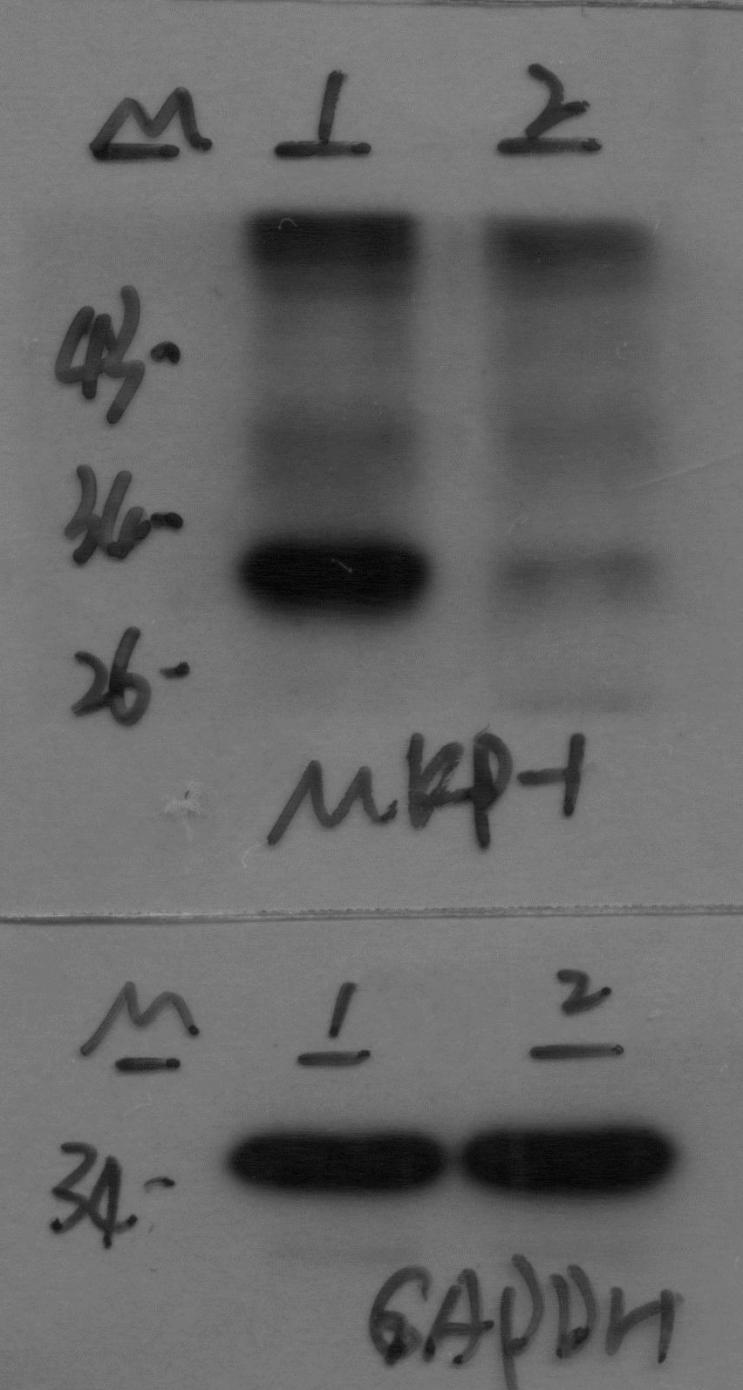


Figure 6: Raw data of FIG 7 JAK2


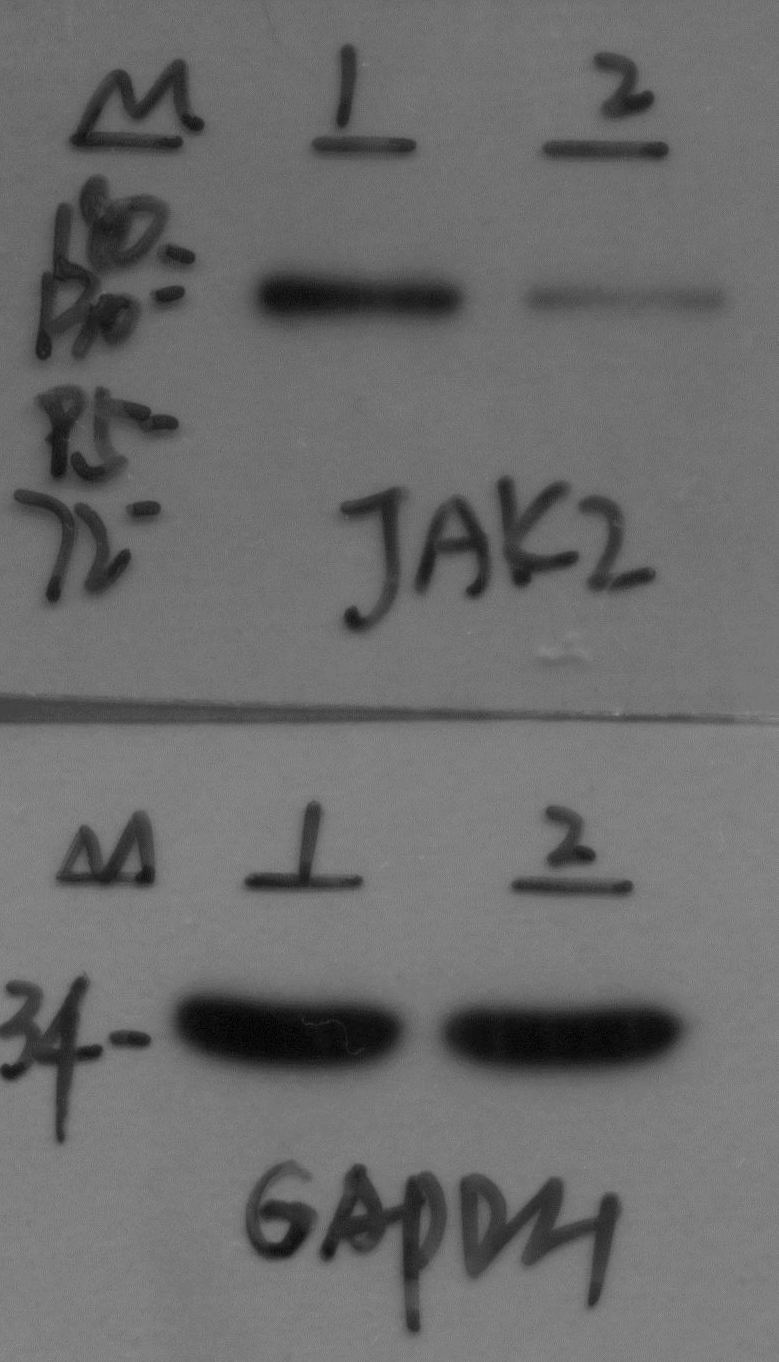


Figure7: Raw data of FIG 7 AKT2


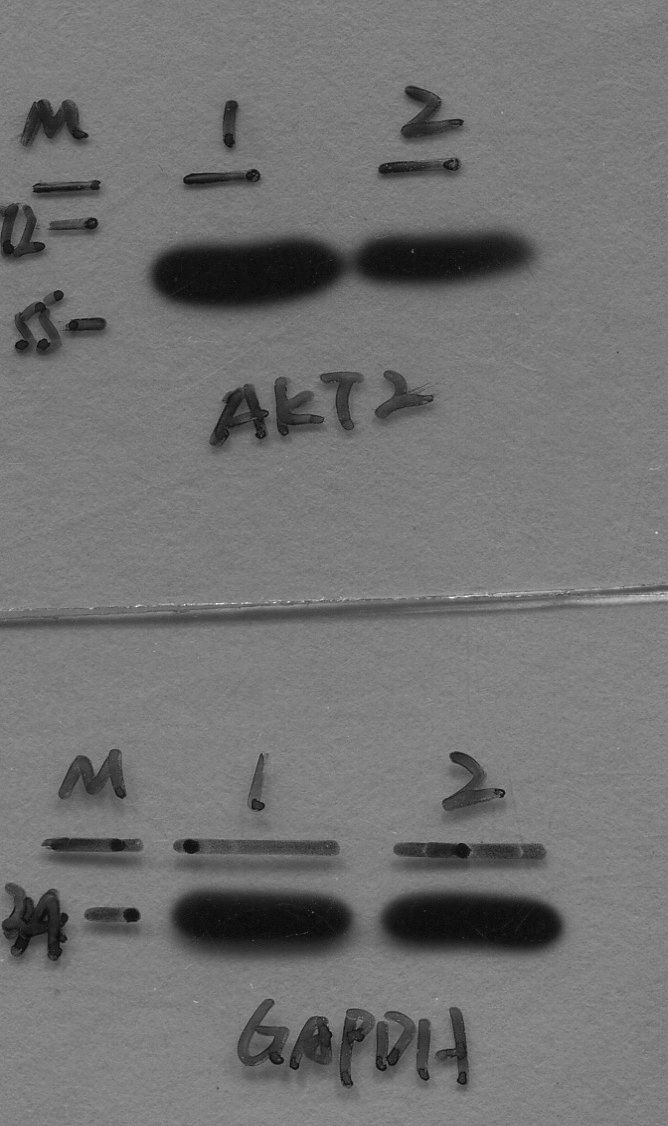


Figure 8: Gray analysis of Figure 1E


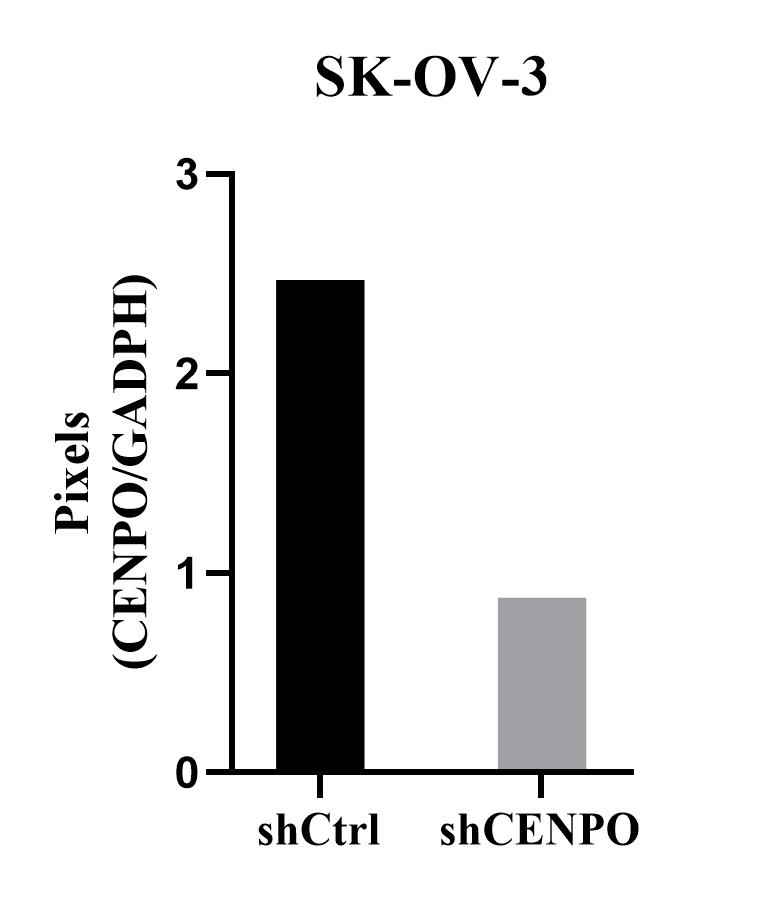

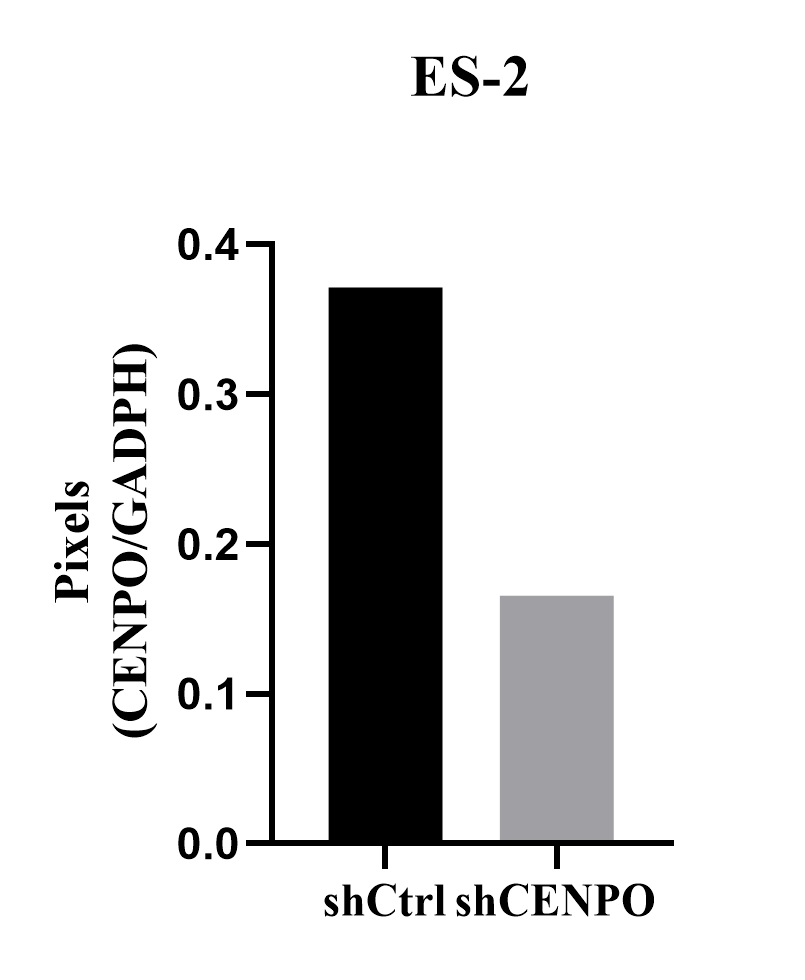


Figure 9: The original diagram of the clone formation experiment


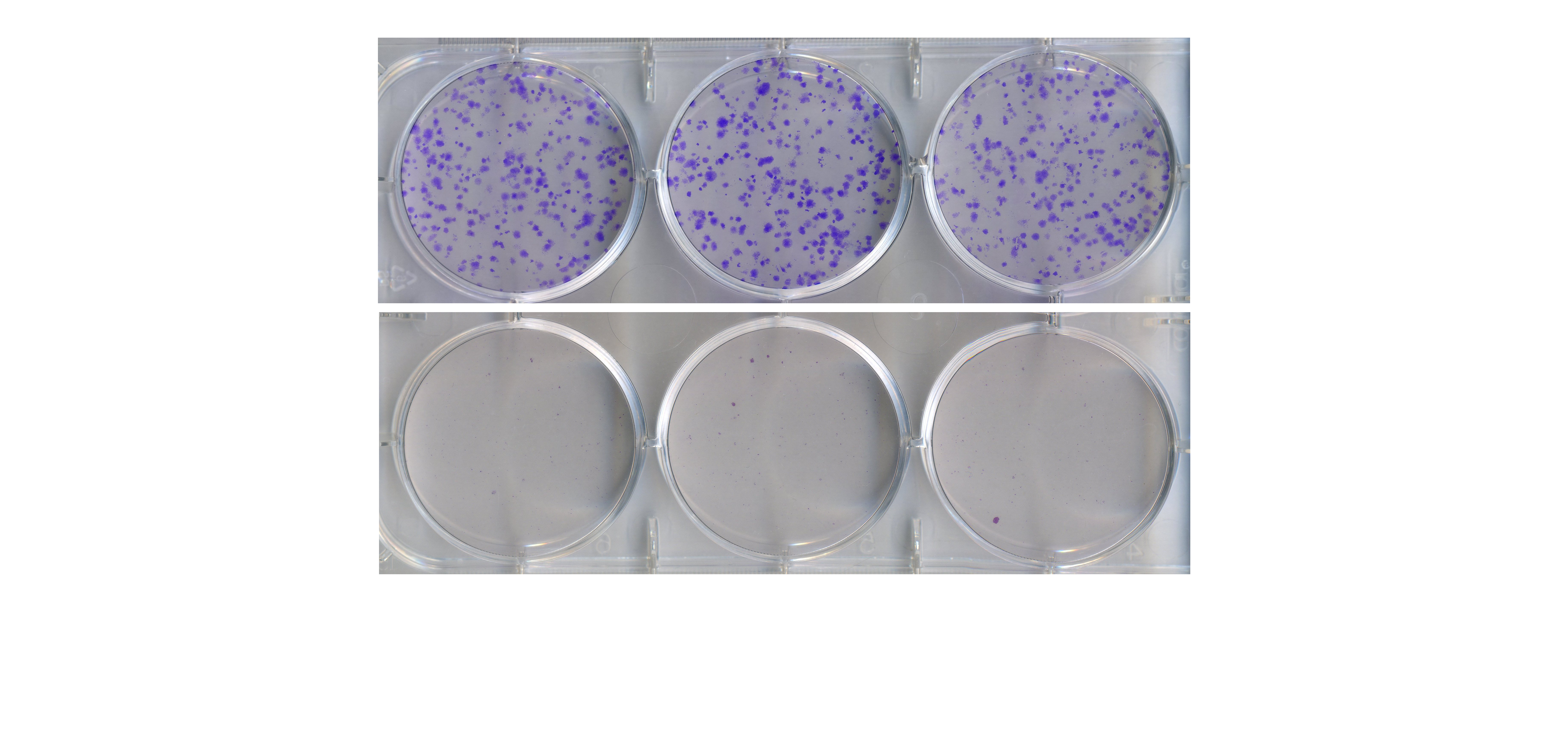

Supplement: Supplementary file 1 — Supplementary Material 1 [file 13048_2024_1452_MOESM1_ESM.docx]
